# BMP signaling in telencephalic neural cell specification and maturation

**DOI:** 10.3389/fncel.2013.00087

**Published:** 2013-06-04

**Authors:** Beatriz Gámez, Edgardo Rodriguez-Carballo, Francesc Ventura

**Affiliations:** Departament de Ciències Fisiològiques II, Institut d’Investigació Biomèdica de Bellvitge, Universitat de Barcelona, L’Hospitalet de LlobregatSpain

**Keywords:** BMP, neural differentiation, morphogen, synaptogenesis, neural development, signal transduction

## Abstract

Bone morphogenetic proteins (BMPs) make up a family of morphogens that are critical for patterning, development, and function of the central and peripheral nervous system. Their effects on neural cells are pleiotropic and highly dynamic depending on the stage of development and the local niche. Neural cells display a broad expression profile of BMP ligands, receptors, and transducer molecules. Moreover, interactions of BMP signaling with other incoming morphogens and signaling pathways are crucial for most of these processes. The key role of BMP signaling suggests that it includes many regulatory mechanisms that restrict BMP activity both temporally and spatially. BMPs affect neural cell fate specification in a dynamic fashion. Initially they inhibit proliferation of neural precursors and promote the first steps in neuronal differentiation. Later on, BMP signaling effects switch from neuronal induction to promotion of astroglial identity and inhibition of neuronal or oligodendroglial lineage commitment. Furthermore, in postmitotic cells, BMPs regulate cell survival and death, to modulate neuronal subtype specification, promote dendritic and axonal growth and induce synapse formation and stabilization. In this review, we examine the canonical and non-canonical mechanisms of BMP signal transduction. Moreover, we focus on the specific role of BMPs in the nervous system including their ability to regulate neural stem cell proliferation, self-renewal, lineage specification, and neuronal function.

## INTRODUCTION

Bone morphogenetic proteins (BMPs) are members of the transforming growth factor β (TGF-β) superfamily (Derynck and Zhang, 2003; Shi and Massague, 2003; Miyazono et al., 2010). BMPs were originally identified as factors that induce bone formation when implanted at ectopic sites (Urist, 1965). Later, it was shown that BMP functions exist in vertebrates as well as in invertebrates and that they perform a wide range of biological action in various cell types and tissues (Chen et al., 2004). BMPs control many indispensable steps in embryogenesis, including the formation and differentiation of the vertebrate nervous system (Mehler et al., 1997; Kishigami and Mishina, 2005; Liu and Niswander, 2005). At initial steps of development, BMP inhibition is required to establish neuroectoderm from ectoderm, although certain levels of BMP signaling are later required for neural crest induction, neural crest cell migration, and spinal cord patterning. At later development stages, BMP signaling promotes astrogliogenesis and inhibition of neuronal or oligodendroglial lineage commitment. Given the functions of BMPs in nervous system development and maintenance, BMP signaling dysfunction and modulation could have a strong impact on various nervous system pathologies as well as their repair processes (Matsuura et al., 2008; Sabo et al., 2009; Ma et al., 2011). In this review, we highlight the main aspects of BMP signaling and BMP’s involvement in neural induction and patterning, embryonic and postnatal neuronal differentiation, and the establishment of neuronal connections.

## SIGNALING BY BMPs

Bone morphogenetic proteins are the largest subfamily of the TGF-β superfamily, which includes 33 members in mammals. BMPs can be further classified into at least four subgroups: BMP-2/4 group, BMP-5/6/7/8 group, growth and differentiation factor-5,-6,-7 (GDF-5/6/7) group, and BMP-9/10 group (Little and Mullins, 2009; Miyazono et al., 2010; Wagner et al., 2010). BMPs are known to be involved in many developmental and homeostatic processes throughout life. However, the exact function of individual BMPs in a particular tissue and at a specific time during development is far from clear. Due to their pleiotropic roles, genetic manipulation often leads to embryonic lethality, thus precluding analysis of their later embryonic or postnatal functions in multiple tissues and organs (Bragdon et al., 2011). In addition, compensatory functional overlaps between BMPs make interpretation complicated. Furthermore, although we may have a rough estimate of the place and the timing of expression of a particular BMP, many factors present in the extracellular environment are able to modify its exact diffusion rate, morphogen range, and its bioavailability for a target cell (Eldar et al., 2002; Peluso et al., 2011).

### BMP SECRETION AND EXTRACELLULAR REGULATION

Bone morphogenetic proteins are synthesized as large precursor proteins comprising an N-terminal signal peptide, which directs the protein to the secretory pathway, a prodomain for proper folding and a C-terminal mature peptide (Sieber et al., 2009). BMPs are first secreted and then proteolitically cleaved upon dimerization by serine proteases such as furin or PC6, releasing the mature peptide (Cui et al., 1998). Active BMPs consist of two monomers stabilized by three intramolecular disulfide bonds, known as cysteine knots, and an inter-dimer disulfide bond (Scheufler et al., 1999). Mature forms may be either homo- or hetero-dimers consisting of different BMP gene products. It has been shown *in vivo* and *in vitro* that some hetero-dimerization could lead to increased functional activity (Valera et al., 2010). In some cases, the cleaved prodomain remains attached to the mature form, as in the case of TGF-β, leading to reduced bioavailability and retention in the extracellular matrix (Ramel and Hill, 2012).

**FIGURE 1:**
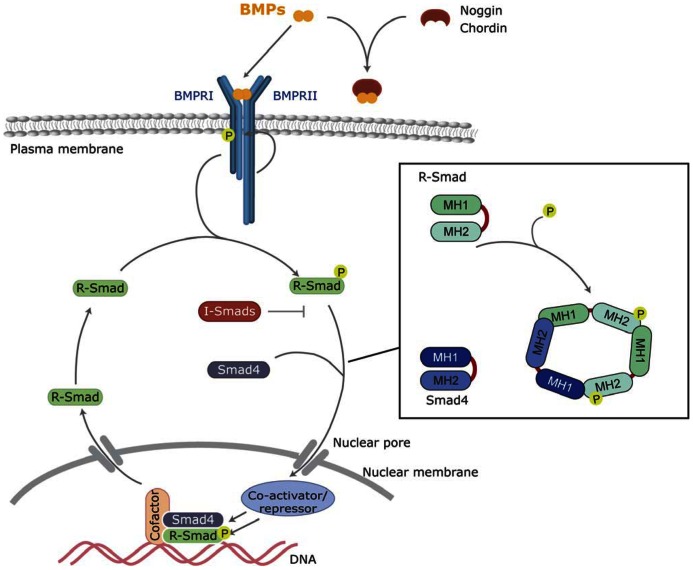
**Canonical BMP signaling**. BMPs bind to the BMP receptors type I and II, and then type II receptor phosphorylates and activates the type I BMP receptor. Activated type I receptor phosphorylates R-Smads, which associate with the common Smad (Smad4) and enter the nucleus, where they regulate transcriptional processes. BMP signaling can be inhibited by extracellular antagonists, such as Noggin and Chordin, or intracellularly by I-Smads.

This restricted availability to their membrane receptors is mostly emphasized by the existence of highly regulated diffusible and cell surface-bound antagonists. There are more than a dozen diffusible antagonists that include chordin, noggin, follistatin, and chordin-like proteins (Rider and Mulloy, 2010; Walsh et al., 2010; Zakin and De Robertis, 2010). Binding of antagonists physically prevents BMPs from binding to their cognate receptors by masking the epitopes involved in ligand–receptor interactions (Groppe et al., 2002; **Figure 1**). Subsequent cleavage of chordin by tolloid zinc metalloproteinases triggers the release of active BMPs from the chordin/BMP complex (Peluso et al., 2011). Twisted gastrulation (Tsg) has a dual role in distinct model systems, acting as either a BMP antagonist or as an agonist. In the case of chordin, the stability of the chordin/BMP complex is greatly increased by *Tsg* (Chang et al., 2001; Ross et al., 2001).

Finally, regulation of BMP transport is crucial for its role as a morphogen. It has been shown that BMP-2 has the ability to link directly to heparan sulfate proteoglycans (HSPGs). In several experimental models, extracellular HSPGs and collagen IV have been involved in the regulation of BMP transport (Ruppert et al., 1996; Osses et al., 2006). Collagen IV binds to BMP-4 and chordin homologs, sequestering them in the extracellular matrix. As mentioned above, Tsg, acting as a BMP agonist, releases chordin/BMP complexes from the collagen IV matrix, allowing their diffusion (Sawala et al., 2012). Thus, all these events of binding of BMPs to the extracellular matrix and/or to antagonists allow the formation of specific gradients through regulated solubility and bioavailability and constitute the first level of signal modulation.

### BMP RECEPTORS AND RECEPTOR ACTIVATION

**FIGURE 2:**
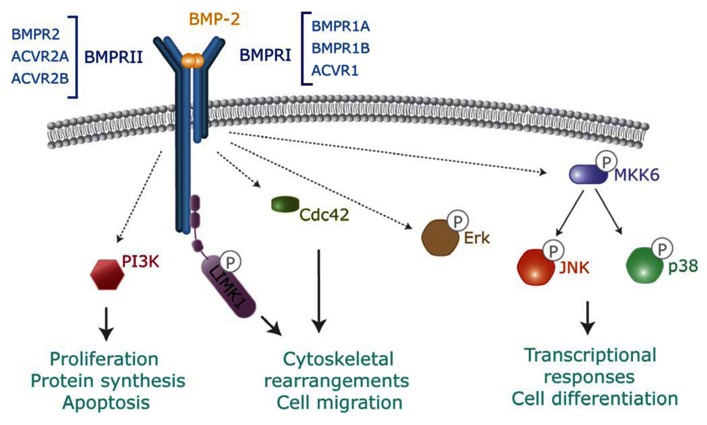
**Non-canonical BMP signaling**. In addition to Smads, activated BMP receptors activate several intracellular pathways which modulate BMP-dependent cellular responses. Pathways include TAK-p38, PI3-kinase, Cdc42 or activation of LIMK by binding to the BMPR-II cytoplasmic tail.

Bone morphogenetic proteins bind to a heterotetrameric complex of transmembrane receptors known as type I and II serine/threonine kinase receptors (Mueller and Nickel, 2012). Both types of receptors contain an N-terminal extracellular binding domain, a single transmembrane region, and an intracellular serine/threonine kinase domain (Shi and Massague, 2003; Feng and Derynck, 2005; Miyazono et al., 2010). Strong evidence confirms that both type I and II receptors, acting in coordination, are required for complete signal transduction. BMPs can bind to type I in the absence of type II receptors but when both types are present in the membrane of target cells their binding affinity is highly increased (Hinck, 2012; **Figure 2**). Based on their structures and functions, type I BMP receptors can be divided into the Bmpr1A and Bmpr1B group (also known as Alk3 and Alk6) and the Acvrl1 and Acvr1 group (also known as Alk1 and Alk2; Hinck, 2012). These groups show slight preferences for binding to specific BMPs. For instance, BMP-2 and -4 bind preferentially Bmpr1A and Bmpr1B whereas BMP-5, -6, and -7 additionally bind to Acvr1 (Liu et al., 1995). It is also well established that BMPs bind to three distinct type II receptors, namely Bmpr2, Acvr2A, and Acvr2B (**Figure 2**). Bmpr2 shows a unique, long C-terminal extension that allows specific recruitment of intracellular transducers. A question arises as to how such a limited number of signaling receptors allows binding of a large number of ligand members. One simple answer would rely on promiscuous interactions between shared ligands and several receptors (Hinck, 2012; Mueller and Nickel, 2012). However, combinatorial interactions of different type I and II receptors should allow selectivity and specificity of ligand binding as well as intracellular signaling in target cells. Numerous *in vivo* examples confirm the unique functions for an individual BMPs that are not shared even for closely related ligands with similar receptor binding affinities *in vitro* (Saremba et al., 2008; Meynard et al., 2009; Perron and Dodd, 2011, 2012). Further study into the molecular mechanisms that drive such functional specificity in living organisms is needed.

Moreover, co-receptors have been shown to modulate ligand binding and downstream signaling events. Members of the repulsive guidance molecule (RGM) family are glycophosphatidylinositol (GPI)-anchored co-receptors for BMP-2 and -4 and enhance responses at low BMP concentrations (Xia et al., 2007). Dragon and hemojuvelin (RGMb and c, respectively) also interact with BMP receptors and ligands and enhance responses for BMP-2 and -6. It has been shown that hemojuvelin plays a major role in regulation of iron metabolism in hepatocytes by increasing the binding affinity of BMP receptors for BMP-6 (Babitt et al., 2006). In addition to facilitating co-receptors, other membrane proteins function as suppressors of BMP signaling. For instance, Bambi is a transmembrane protein, structurally related to type I receptors that act as pseudoreceptor. Bambi forms a stable receptor complex but, since it lacks the intracellular kinase domain, inhibits transduction by titrating available type II receptors (Onichtchouk et al., 1999).

Type I and II BMP receptors show some ligand-independent affinity for each other. In the absence of a ligand, small amounts of pre-existing homo- and hetero-dimer receptor complexes are present at the cell surface (Ehrlich et al., 2012). However, ligand binding dramatically increases oligomerization involving type I and II complexes. Ligand-induced oligomerization promotes type II-dependent phosphorylation of a specific domain of type I receptors (known as the GS domain; **Figure 1**). Upon phosphorylation of the GS domain the type I receptor kinase is converted to its active conformation (Wrana et al., 1994). Thus, the kinase activity of type I receptors is the major intracellular transducer: whereas mutated or kinase-deficient type I receptors block most of the cellular responses to the ligand, constitutively active type I receptors (induced by pathological mutations or artificially designed) are able to signal most of the responses in the absence of type II receptors or ligands (Wieser et al., 1995).

### INTRACELLULAR Smad SIGNALING FROM BMP RECEPTORS

Smads are the main and best-known intracellular signal transducers for BMP receptors. According to their structural and functional properties, three different types of Smads have been defined: the receptor-regulated Smads (R-Smad) Smad1, -2,-3,-5, and -8; a common mediator Smad, Smad4; and the inhibitory Smads Smad6 and -7 (Shi and Massague, 2003; Sieber et al., 2009; Miyazono et al., 2010). Active type I kinases phosphorylate R-Smads at serine residues located in their C-terminus. Specific phosphorylation of different R-Smads depends on the L45 loop of the type I receptor (Feng and Derynck, 1997). All the BMP type I receptors mentioned above (BMPR1A, BMPR1B, Acvr1, and Acvrl1) phosphorylate Smad1, Smad5, and Smad8, which are thus defined as BMP-activated Smads. Phosphorylation and activation of R-Smads disrupts the autoinhibitory interaction between the N-terminal (MAD homology 1, MH1) and C-terminal (MH2) domains of Smad monomers (Shi et al., 1997). This favors the formation of a multimeric complex composed of two molecules of R-Smad and one molecule of Smad4 interacting through their MH2 domain (**Figure 1**). On the new conformation, the nuclear import signal is exposed and the complexes translocate into the nucleus where they execute distinct transcriptional regulatory functions (Feng and Derynck, 2005; Hill, 2009).

Intracellular BMP signaling, as shown for transduction of all other morphogens described so far, is subjected to a growing number of cross-talk mechanisms with other extracellular ones, as well as regulation by internal cues, in order to integrate a final cell response. For instance, the inhibitory Smads, Smad6, and Smad7, block BMP signaling by preventing phosphorylation of R-Smads by type I receptors in a dominant negative fashion by binding to active receptor complexes (Derynck and Zhang, 2003). Another known mechanism is the degradation of Smads through the ubiquitin–proteasome pathway. Several homologous to E6-associated protein C-terminus (HECT)-type E3 ligases, such as Smurf-1,-2, or Nedd4-2, interact and ubiquitinate Smads and, when complexed with I-Smads, BMP receptors (Wicks et al., 2006). Degradation of Smads is also regulated by mitogen-activated protein (MAP) kinase and Gsk-3 phosphorylation allowing specific interaction with MAP kinase and Wnt signaling cascades (Fuentealba et al., 2007).

### NON-CANONICAL SIGNALING FROM BMP RECEPTORS

In addition to Smads, BMPs activate other intracellular signaling pathways relevant to their cell functions. Non-canonical BMP signaling includes Rho-like small GTPases, phosphatidylinositol 3-kinase/Akt (PI3K/Akt) or various types of MAP kinases (Derynck and Zhang, 2003; Zhang, 2009; **Figure 2**). Mechanistically, it is well established that BMPs regulate the Tak1/p38 pathway through recruitment and ubiquitylation of Traf6 by activated receptor complexes (Sorrentino et al., 2008; Yamashita et al., 2008). BMPs trigger canonical and non-canonical pathways simultaneously, driving to a specific cellular output (Ulsamer et al., 2008; Xu et al., 2008; Susperregui et al., 2011). Several studies indicate that BMP-mediated cell migration or axon and dendrite growth requires activation of the small GTPase Cdc42 and Limk activities. Most of these effects are Smad-independent, but depend on Limk binding to the long cytoplasmatic tail of Bmpr2 complexes (Foletta et al., 2003; Lee-Hoeflich et al., 2004). Furthermore, additional reports indicate that activation of Limk also requires the activation of Paks through Cdc42 and PI3K, as well as p38 MAP kinase activities (Gamell et al., 2008, 2011). The specific abilities of distinct receptors or receptor combinations to activate these non-canonical pathways and promote specific signaling outcomes need to be studied.

## BMPs IN NERVOUS SYSTEM DEVELOPMENT AND MAINTENANCE

**FIGURE 3:**
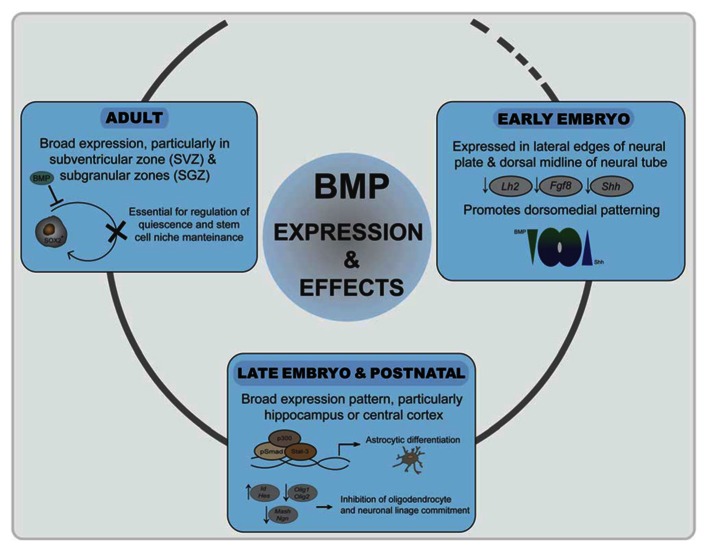
**Roles of BMP signaling in neural development**. Initially, inhibition of BMP signaling is required for neuroectoderm induction. BMPs generate a signaling gradient and promote dorsomedial identity. BMP signaling is prominent at the dorsal telencephalic midline and in the marginal zone neurons, where it correlates negatively with expression of the cortical selector *Lhx2*, *Fgf8*, or *Shh.* In late embryogenesis and postnatally, BMP signaling promotes astroglial commitment by activation astrocyte-specific promoters through a Stat3-p300/CBP-Smad1 complex. At the same time represses commitment to the other two neural lineages by inducing Ids and Hes proteins. In adulthood, a balanced BMP signaling is required for regulation of quiescence and differentiation of neural stem cell populations in the SVZ and SGZ.

In recent years, there has been exponential progress in the clarification of the role of BMPs at different stages of nervous system development. BMP ligands and receptors are expressed in very complex patterns throughout neural development in all regions of the central and peripheral nervous system (Ebendal et al., 1998; Miyagi et al., 2011). Evidence clearly demonstrates that these pleiotropic cytokines have a very dynamic role: from the early steps of neuralization and patterning to an instructive role in neural precursor fate commitment and neuronal wiring (Liu and Niswander, 2005). Evidence also extends their role in postnatal brain as well as peripheral nervous system development and maintenance (Bond et al., 2012; **Figure 3**).

### EXPRESSION AND ROLE OF BMPs AND RECEPTORS IN TELENCEPHALIC TISSUES

Bone morphogenetic proteins and their antagonists are expressed throughout neural development with a complex chronological and overlapping location that is only known to a limited extent. In early-developing neural tissue, BMPs are expressed particularly at the lateral edges of the neural plate and later on in the dorsal midline of the neural tube (Mehler et al., 1997; Chen and Panchision, 2007). During vertebrate development, the rostral–dorsal part of the neural plate gives rise to the telencephalon, the most complex region of the nervous system. The two signaling centers in the dorsal midline of the telencephalon, the roof plate and the cortical hem, secrete several BMPs (Furuta et al., 1997). These BMPs generate a signaling gradient and promote dorsomedial identity. Mice that are transgenic for specific BMP-dependent reporters indicate that, at embryonic stages, high BMP signaling is prominent at the dorsal telencephalic midline and in the marginal zone neurons, where it correlates negatively with expression of the cortical selector *Lhx2* (Doan et al., 2012). BMPs also reduce the expression of the anterior forebrain markers *Fgf8* or *Shh* (Anderson et al., 2002; **Figure 3**). *Chordin* and *noggin* double mutant embryos further emphasize the importance of the appeasement of BMP signaling for proper ventral forebrain development (Anderson et al., 2002). During further embryo development, brain expression of BMP-2, -4, -5, -6, and -7 peaks around postnatal day 4 with a broad expression pattern (Mehler et al., 1997; Sabo et al., 2009). Particular areas of the telencephalon, such as the hippocampus or cerebral cortex, show strong postnatal BMP activation (Doan et al., 2012). In adult telencephalon, there is also broad expression of BMPs at most locations and cell types, suggesting also a pivotal role in the adult brain. Interestingly, expression of BMPs and their antagonists specifically remain in the two areas where neurogenesis continues in the adult [the subventricular zone (SVZ) and subgranular zones (SGZ); Bond et al., 2012]. Expression of BMPs, from stem cells and neural progenitors, and noggin, secreted from ependymal cells, is essential for stem cell niche maintenance and neuroblast survival (Lim et al., 2000; Mira et al., 2010; **Figure 3**). Moreover, increased expression of BMPs occurs after distinct brain and spinal cord injuries and suggest a role of BMP signaling in neural cell survival and repair (Sabo et al., 2009).

Bone morphogenetic protein receptors are expressed at high levels throughout all stages of embryonic development but show different expression patterns. *Bmpr1A* is expressed earlier than *Bmpr1B*. *Bmpr2* is mostly restricted to proliferative regions of the nervous system, whereas *Acvr2* and *Acvr1* have also been detected in early neural development and are expressed at high levels in adult brain. Bmpr1A and Bmpr1B are required separately in some development processes although in several studies of neural development, each receptor could, at least partially, compensate for the loss of the other. For instance, deletion of *Bmpr1B* causes no obvious forebrain phenotype (Yi et al., 2000). However, the requirement of BMP signaling for dorsal telencephalic development is shown after forebrain-specific ablation of *Bmpr1A* or the double knock-out of *Bmpr1A* and *Bmpr1B*, which leads to holoprosencephaly (Fernandes et al., 2007). However, later ablation only affects formation of the dentate gyrus of the hippocampus (Caronia et al., 2010). In adult telencephalon, expression of all these receptors remains, with *Bmpr1A* the most abundant and broadly expressed one (Miyagi et al., 2011). All cell types (neurons, astroglia, oligodendroglia, or ependymal cells) express combinations of these receptors (Chalazonitis et al., 2011; Bond et al., 2012). Interestingly, some reports suggest that their expression pattern is differentially distributed within a single neuron, with Bmpr1A mainly expressed in cell bodies and Bmpr1B in dendrites (Miyagi et al., 2011).

### BMPs IN EMBRYONIC AND ADULT NEURAL CELL SPECIFICATION

The central and peripheral nervous systems originate from neural progenitor cells, which proliferate and give rise to the three main neural cell types: neurons, astrocytes, and oligodendrocytes. The specification and differentiation of the distinct cell types require interactions between cell-autonomous molecular mechanisms and external signaling cues (Mehler et al., 1997; Mehler, 2002; Liu and Niswander, 2005). Remarkably, BMPs are critical for progenitor cell specification and maintenance of a particular phenotype through dynamic transcriptional regulation (Bond et al., 2012). BMPs decrease proliferation of neural progenitors in cell culture models as well as *in vivo* in combination with other signaling molecules and internal cues (Chmielnicki et al., 2004; Sun et al., 2011; Benraiss et al., 2012).

#### BMP role in neurogenesis

**FIGURE 4:**
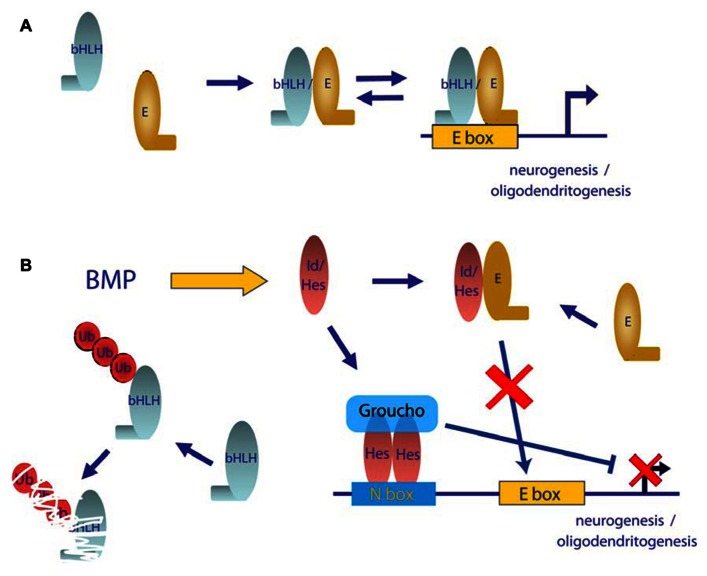
**Model of mechanism of repression of neurogenic bHLH transcription factors by BMPs**. **(A)** In differentiating neurogenic precursors, bHLH transcription factors hetero-dimerizes with E proteins, which bind to E boxes and promote expression of neuronal genes. **(B)** Activation of the BMP signaling pathway leads to increased levels of Id and Hes proteins. Id and Hes proteins sequester E proteins away from bHLH transcription factors, which leads to transcriptionally inactive complexes and also enhance degradation of bHLH monomers. Furthermore Hes proteins can bind to N boxes sequences in neural promoters and recruit the Groucho family of transcriptional repressors.

Neurogenesis is promoted by proneuronal basic helix-loop-helix (bHLH) transcription factors including Mash1, Neurogenin, or NeuroD, which form hetero-dimers with ubiquitously expressed bHLH E proteins, such as the E2A gene products, E12 and E47, through their HLH domain (Ross et al., 2003; Hsieh, 2012). Hetero-dimers bind to DNA through their basic domain and activate the transcription of genes that have E boxes in their promoter region (**Figure 4A**). One subfamily of HLH factors, known as Id proteins, lacks this basic region. Hetero-dimerization of Id with bHLH is sufficient to block their DNA binding and function (Norton, 2000; Ruzinova and Benezra, 2003). Moreover, Ids not only inhibit transcriptional function but also promote the degradation of neurogenic bHLH by sequestering ubiquitous E proteins (Vinals et al., 2004; **Figure 4B**). Similarly, the Hes family of bHLH transcriptional repressors blocks neuronal differentiation through a dominant negative function on E protein availability as well as through direct binding on specific promoters and recruitment of members of the Grouch family of transcriptional repressors (**Figure 4B**). BMPs have been shown to be strong inducers of both *Id* and *Hes* family members (Ross et al., 2003). Thus, Id and Hes family members are thought to be major molecular players in the negative effects of BMPs on commitment and differentiation of neuronal precursors (Takizawa et al., 2003; Imayoshi et al., 2008). Furthermore, BMPs have been proved to increase the expression of the transcriptional repressor *Rest/Nsrf*. Expression of *Rest/Nsrf* allows continuous repression of the neuronal markers in cells committed to other lineages (Kohyama et al., 2010). In addition to their effects on embryonic neurogenesis, BMPs are widely accepted as relevant molecules in adult neurogenesis. In adult telencephalon, neural stem cell populations are maintained in two niches: the SVZ and the SGZ of the dentate gyrus. Colak et al. (2008) showed, through conditional deletion of *Smad4* or noggin infusion, that BMP signaling was required for adult neurogenesis. Noggin can also expand hippocampal progenitors in the SGZ (Bonaguidi et al., 2008). Infusion of Noggin and genetic deletion of either *Bmpr1A* or *Smad4* further demonstrate the role of BMP signaling in regulation of quiescence of neural stem cells from SGZ, restraining their proliferation and allowing these niches to maintain long-term neurogenic ability (Mira et al., 2010). BMP signaling is also required for positional identity and neuronal subtype specification. Endogenously produced BMPs inhibit the expression of a telencephalic gene profile, which was revealed by addition of noggin or other pharmacological signaling inhibitors (Bertacchi et al., 2013). BMPs are also involved in regulation of a transcriptional network to generate forebrain cholinergic neurons (Bissonnette et al., 2011). They also determine a temporally and spatially restricted requirement for generation of glutamatergic neurons in cerebellum (Fernandes et al., 2012).

#### BMP role in oligodendrogenesis

Oligodendrocyte specification and maturation depend on the function of transcription factors that include Olig1, Olig2, and Sox10. Olig2 directs early oligodendrocyte specification and Olig1 promotes oligodendrocyte maturation and is required for repair of demyelinated lesions (Lu et al., 2002; Ligon et al., 2006; Li and Richardson, 2008). BMPs inhibit the development of several stages of oligodendrocyte differentiation as well as the timing of myelination as shown by expression analysis markers of oligodendrocyte differentiation such as A2B5, galactocerebroside, or myelin protein expression (Hall and Miller, 2004; Samanta and Kessler, 2004; Chen and Panchision, 2007; See and Grinspan, 2009; Weng et al., 2012). Conversely, noggin blocks production of astrocytes from oligodendrocyte precursors in culture (Sim et al., 2006). BMP-4 stimulation increases oligodendrocyte progenitor proliferation in a model of induced demyelination. More importantly, addition of noggin increases the number of mature oligodendrocytes and increased the remyelination of injured axons in the corpus callosum (Sabo et al., 2011; Wu et al., 2012; Sabo and Cate, 2013). At the molecular level, BMP-induced expression of Id and Hes proteins seems to be also relevant in such a process by sequestering Olig1 and Olig2 which also belong to the bHLH family of transcription factors (Cheng et al., 2007; Bilican et al., 2008). Additional targets of Notch, such as *Jag1*, *Hey1*, and *Hey2*, are upregulated by BMP in oligodendrocytes through increased epigenetic modification at these loci (Wu et al., 2012). Inhibition of *Olig2* expression by direct binding of Smad4 to the *Olig2* promoter has also been demonstrated (Bilican et al., 2008; **Figure 3**). Recently, it has also been shown that the Smad-interacting protein-1 (Sip1) is an essential *in vivo* modulator of myelination. Sip1 antagonizes BMP signaling acting as a transcriptional repressor of Smad activity, while promoting activation of Olig1/Olig2 transcriptional activity and induction of I-Smads (Weng et al., 2012).

#### BMP role in astrocytogenesis

In opposition to the repression of neuronal and oligodendrocyte development, during the late embryonic and postnatal periods, BMP signaling strongly induces astrocyte differentiation (Gross et al., 1996; Mehler et al., 1997; Fukuda et al., 2007; See et al., 2007). BMPs promote the generation of astrocytes from precursors in a variety of embryonic neural cells (Gross et al., 1996; Mehler et al., 1997; Mehler, 2002; Bonaguidi et al., 2005). Implantation of noggin-producing cells induced the appearance of increased numbers of oligodendrocyte precursors whereas high BMP signaling inhibits oligodendrocyte precursors and promote astrogliogenesis (Mabie et al., 1999; Mekki-Dauriac et al., 2002; Wu et al., 2012). BMP signaling in the SVZ promotes astroglial lineage commitment and block differentiation of neurons and oligodendrocytes, whereas noggin suppresses astrocyte determination (Chmielnicki et al., 2004; Colak et al., 2008). LIF and BMPs synergize to promote astrocytic differentiation by activating astrocyte-specific promoters through a Stat3-p300/CBP-Smad1 complex (Nakashima et al., 1999; **Figure 3**). The ability of BMPs and Stat3 to promote astrogliogenesis has been shown to be dependent on the histone acetylation/deacetylation machinery (Scholl et al., 2012). Another study indicated that Smad action is not required for gliogenesis but is promoted by BMPs through mammalian target of rapamycin/FKBP12-rapamycin-associated protein (mTor/Frap) phosphorylation of Stats (Rajan et al., 2003). Interestingly, Bmpr1A or Bmpr1B signaling exerts opposing effects on initial astrocytic hypertrophy after injury, suggesting that distinct BMPs engaging different receptor complexes exert separate activities on gliogenesis. The ability of BMPs to promote maturation of astroglia is further emphasized by the fact that activation of BMP signaling inhibits the tumorigenic potential of human glioblastoma-initiating cells (Piccirillo et al., 2006). Furthermore, Ezh2-dependent epigenetic silencing of *Bmpr1B* desensitizes tumor-initiating glioblastoma cells to differentiation and contributes to their tumorigenicity (Lee et al., 2008).

### BMP SIGNALING IN NEURITE AND AXON OUTGROWTH, GUIDANCE AND SYNAPSE FORMATION

After neurogenesis has been completed, several sequential events are needed to establish neuronal circuits: polarized outgrowth of axons and dendrites, axon path finding toward the appropriate synaptic partner cell and establishment and maintenance of the synapse. Growing evidence in several experimental models points to BMP regulation of all these events. For instance, BMP ligands display positive regulation of the number, length, and branching of neurites in neurons from diverse neuronal origins, including cortical and hippocampal neurons (Le Roux et al., 1999; Withers et al., 2000; Lee-Hoeflich et al., 2004; Podkowa et al., 2010). Similarly, BMPs have been included as inductive signals promoting growth cone guidance as well as axonal orientation and path finding. Most of the data arise from studies of the sensory projection neurons of the spinal cord. BMP-7 and GDF-7 supplied by the roof plate orient axons of commissural neurons away from the roof plate and regulate their rate of extension through the spinal cord toward the floor plate (Augsburger et al., 1999; Butler and Dodd, 2003; Yamauchi et al., 2008, 2013; Sanchez-Camacho and Bovolenta, 2009). Guideposts are discrete groups of glial or neuronal cells that provide discontinuous information in intermediate positions along the path of growing axons (Sanchez-Camacho and Bovolenta, 2009). The corpus callosum represents the major forebrain commissure connecting the two cerebral hemispheres. Midline crossing of callosal axons is controlled by several glial and neuronal guideposts specifically located along the callosal path. BMP-7 has been shown to be required at different steps of organization and differentiation of these guidepost cells, which allows formation of the corpus callosum (Sanchez-Camacho et al., 2011). Additional evidence also indicates that BMP-7, secreted from the meninges, is involved in a morphogenic cascade, including Wnt3a and GDF-5, allowing correct corpus callosum development and prevents premature axon projection (Choe et al., 2012). It is not clear which signaling mechanisms are activated by BMP in these processes. In contrast to the slow timing of neural and glial specification, the very rapid time course of BMP-induced axonal orientation suggests transcription-independent pathways, probably depending on cytoskeletal actin remodeling and c-Jun N-terminal kinase (Jnk)-mediated microtubule stabilization (Augsburger et al., 1999; Wen et al., 2007; Podkowa et al., 2010; Perron and Dodd, 2011). Regulation of the cytoskeleton by BMPs has been linked to activation of Limk1 activation. Limk1 and Limk2 are closely related kinases that phosphorylate and inactivate actin-depolymerizing proteins such as cofilin or ADF. Limk1 has been shown to be activated by several BMPs in neural cells through specific binding to the Bmpr2 cytoplasmic tail and a further activation mechanism that involves Rho GTPases and PI3-kinase (Foletta et al., 2003; Lee-Hoeflich et al., 2004; Eaton and Davis, 2005; Gamell et al., 2008). Limk phosphorylation of cofilin/ADF is counteracted by the action of the Slingshot phosphatase, which is activated at later times after BMP addition and enables chemotactic responses to change from attraction to repulsion (Wen et al., 2007).

An intriguing functional and mechanistic question is that, whereas the closely related BMP-6 and -7 both induce the differentiation of commissural neurons, only BMP-7 is able to orients its axons *in vitro* and is required for appropriate axon projection *in vivo* (Perron and Dodd, 2011, 2012). Both ligands have been reported to bind hetero-dimers consisting of Acvr2A or Bmpr2 with Acvr1, Bmpr1A or Bmpr1B in numerous cellular models (Mueller and Nickel, 2012). However, the facts that a single amino acid swapping allows BMP-6 to orient axons and *vice versa* and that binding of BMP-6 to type I receptor depends on N-glycosylation, suggest that distinct receptor recruitment is involved in these functional differences (Saremba et al., 2008; Perron and Dodd, 2012). Changes in expression of BMP receptor subunits at growth cones have been shown after motor neuronal differentiation (Benavente et al., 2012). Moreover, whereas neuronal specification is a redundant function of Bmpr1A and Bmpr1B, axon outgrowth and regulation of cofilin activity only depend on *Bmpr1B* (Yamauchi et al., 2008, 2013).

#### BMP role in synaptogenesis

**FIGURE 5:**
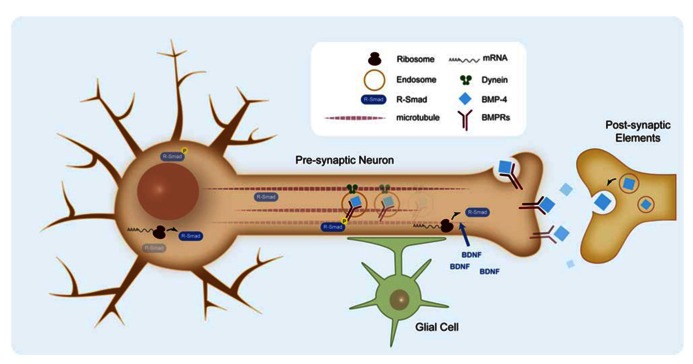
**Retrograde signaling of BMPs in synapse formation**. BMP retrograde transport of endocytosed BMP receptors by dynein motors. Effective retrograde BMP signal also involves specific translation of Smad protein in the axons.

Once axons reach their corresponding target, two-way communication between presynaptic and postsynaptic cells is needed for synaptic establishment and homeostasis during development and for proper synaptic plasticity. The *Drosophila* larval neuromuscular junction (NMJ) is a useful model for genetic and biochemical studies of synaptic function (Collins and DiAntonio, 2007). Development of the synapse requires an anterograde as well as retrograde input from the synaptic terminal and target cell. BMP signaling is an indispensable component of retrograde signaling in the NMJ (Henriquez et al., 2011). The muscle-secreted BMP ligand *glass bottom boat* (*Gbb*), signals through presynaptic *wishful thinking* (*Wit*), the Bmpr2 homolog, and the receptor type I homologs *thick veins* and *saxophone* (*Tkv* and *Sax*, respectively; Aberle et al., 2002; Marques et al., 2002, 2003; Rawson et al., 2003). *Wit* mutant larvae show complete presynaptic detachment, which could be rescued by its expression in presynaptic cells (Aberle et al., 2002; Marques et al., 2002). Similarly, *Gbb* expression in muscle, but not in neurons, rescues NMJ defects observed in *Gbb* mutants (Marques et al., 2003). Retrograde *Gbb* activation of synaptic receptors has two parallel effects. One is activation of Limk1 that allows stabilization of the synapse. In the absence of *Limk1*, synaptic footprints are observed in the NMJ. In these footprints the presynaptic components are missing (Eaton and Davis, 2005). Since presynaptic development precedes postsynaptic development, synaptic footprints have been interpreted as being the remnants of mature synaptic contacts that have formed and then retracted. The other effect involves activation of the Smad homologs *Mad* and *Medea* and their transport to the neuronal soma to regulate transcription at the nucleus (**Figure 5**). There, Mad-dependent transcription of the Rac-guanine nucleotide exchange factor (GEF) *Trio* is relevant for proper NMJ growth and branching since transgenic expression of *Trio* partially rescues BMP signaling mutant larvae (Ball et al., 2010). Therefore, canonical and non-canonical pathways are involved in coordination of the synaptic growth and stability of NMJ. Interestingly, genes related to several motor diseases such as hereditary spastic paraplegia or amyotrophic lateral sclerosis, have been shown to regulate BMP retrograde signaling in model systems (Bayat et al., 2011; Henriquez et al., 2011).

An important aspect in BMP retrograde communication is how signals at the synaptic terminal are conveyed to the nucleus to regulate transcription. It has been shown that BMP retrograde signaling along the axon requires dynein retrograde motors (McCabe et al., 2003). Recently, retrograde transport of endocytosed BMP receptors has been demonstrated, which suggests two populations of phosphorylated Smad transducers, one at the synaptic terminal and one at the cell body (Smith et al., 2012; **Figure 5**). However, studies of the murine trigeminal sensory system indicate that an effective retrograde BMP signal also involves specific translation of Smad protein in the axon, which is transported to the cell body by dynein motors (Ji and Jaffrey, 2012). Axonal translation of Smads is activated by brain-derived neurotrophic factor (BDNF), thus coupling both morphogens in the homeostasis of synapses. Some questions remain about the exact place of Smad activation and the role of the additional Smads translated in the axon when it is commonly accepted that the intracellular pool of unphosphorylated Smads is relatively high. Another relevant aspect is the role of glial cells in the regulation of synaptogenesis. Glia, associate intimately with synaptic terminals and are required for synaptogenesis. Recent work on NMJ points to a new member of the BMP ligand family, *Maverick* (*Mav*), in controlling synthesis and release of *Gbb*. Mav is released in glial cells surrounding the synapse and reinforces BMP retrograde signaling by transcriptional regulation of the synthesis of *Gbb* in postsynaptic cells (Fuentes-Medel et al., 2012). Altogether, evidence points to a very relevant role of BMP ligands in the coordination of several cell types and signaling mechanisms during synaptogenesis.

## Conflict of Interest Statement

The authors declare that the research was conducted in the absence of any commercial or financial relationships that could be construed as a potential conflict of interest.

## References

[B1] Aberle H. Haghighi A. P. Fetter R. D. McCabe B. D. Magalhaes T. R. Goodman C. S. (2002). wishful thinking encodes a BMP type II receptor that regulates synaptic growth in *Drosophila*. *Neuron* 33 545–558.doi: 10.1016/S0896-6273(02)00589-5 11856529 10.1016/s0896-6273(02)00589-5

[B2] Anderson R. M. Lawrence A. R. Stottmann R. W. Bachiller D. Klingensmith J. (2002). Chordin and noggin promote organizing centers of forebrain development in the mouse. *Development* 129 4975–4987 12397106 10.1242/dev.129.21.4975

[B3] Augsburger A. Schuchardt A. Hoskins S. Dodd J. Butler S. (1999). BMPs as mediators of roof plate repulsion of commissural neurons. *Neuron* 24 127–141 10.1016/S0896-6273(00)80827-2 10677032

[B4] Babitt J. L. Huang F. W. Wrighting D. M. Xia Y. Sidis Y. Samad T. A. (2006). Bone morphogenetic protein signaling by hemojuvelin regulates hepcidin expression. *Nat. Genet.* 38 531–539 10.1038/ng1777 16604073

[B5] Ball R. W. Warren-Paquin M. Tsurudome K. Liao E. H. Elazzouzi F. Cavanagh C. (2010). Retrograde BMP signaling controls synaptic growth at the NMJ by regulating trio expression in motor neurons. *Neuron* 66 536–549 10.1016/j.neuron.2010.04.011 20510858

[B6] Bayat V. Jaiswal M. Bellen H. J. (2011). The BMP signaling pathway at the *Drosophila* neuromuscular junction and its links to neurodegenerative diseases. *Curr. Opin. Neurobiol.* 21 182–188 10.1016/j.conb.2010.08.014 20832291 PMC3095363

[B7] Benavente F. Pinto C. Parada M. Henriquez J. P. Osses N. (2012). Bone morphogenetic protein 2 inhibits neurite outgrowth of motor neuron-like NSC-34 cells and up-regulates its type II receptor. *J. Neurochem.* 122 594–604 10.1111/j.1471-4159.2012.07795.x 22612292

[B8] Benraiss A. Bruel-Jungerman E. Lu G. Economides A. N. Davidson B. Goldman S. A. (2012). Sustained induction of neuronal addition to the adult rat neostriatum by AAV4-delivered noggin and BDNF. *Gene Ther.* 19 483–493 10.1038/gt.2011.114 21918547 PMC3655807

[B9] Bertacchi M. Pandolfini L. Murenu E. Viegi A. Capsoni S. Cellerino A. (2013). The positional identity of mouse ES cell-generated neurons is affected by BMP signaling. *Cell. Mol. Life Sci.* 70 1095–1111 10.1007/s00018-012-1182-3 23069989 PMC3578729

[B10] Bilican B. Fiore-Heriche C. Compston A. Allen N. D. Chandran S. (2008). Induction of Olig2 precursors by FGF involves BMP signalling blockade at the Smad level. *PLoS ONE* 3: e2863. 10.1371/journal.pone.0002863 PMC2483937 18682850

[B11] Bissonnette C. J. Lyass L. Bhattacharyya B. J. Belmadani A. Miller R. J. Kessler J. A. (2011). The controlled generation of functional basal forebrain cholinergic neurons from human embryonic stem cells. *Stem Cells* 29 802–811 10.1002/stem.626 21381151 PMC3107131

[B12] Bonaguidi M. A. McGuire T. Hu M. Kan L. Samanta J. Kessler J. A. (2005). LIF and BMP signaling generate separate and discrete types of GFAP-expressing cells. *Development* 132 5503–5514 10.1242/dev.02166 16314487

[B13] Bonaguidi M. A. Peng C. Y. McGuire T. Falciglia G. Gobeske K. T. Czeisler C. (2008). Noggin expands neural stem cells in the adult hippocampus. *J. Neurosci.* 28 9194–9204 10.1523/JNEUROSCI.3314-07.2008 18784300 PMC3651371

[B14] Bond A. M. Bhalala O. G. Kessler J. A. (2012). The dynamic role of bone morphogenetic proteins in neural stem cell fate and maturation. *Dev. Neurobiol.* 72 1068–1084 10.1002/dneu.22022 22489086 PMC3773925

[B15] Bragdon B. Moseychuk O. Saldanha S. King D. Julian J. Nohe A. (2011). Bone morphogenetic proteins: a critical review. *Cell. Signal.* 23 609–620 10.1016/j.cellsig.2010.10.003 20959140

[B16] Butler S. J. Dodd J. (2003). A role for BMP heterodimers in roof plate-mediated repulsion of commissural axons. *Neuron* 38 389–401 10.1016/S0896-6273(03)00254-X 12741987

[B17] Caronia G. Wilcoxon J. Feldman P. Grove E. A. (2010). Bone morphogenetic protein signaling in the developing telencephalon controls formation of the hippocampal dentate gyrus and modifies fear-related behavior. *J. Neurosci.* 30 6291–6301 10.1523/JNEUROSCI.0550-10.2010 20445055 PMC2905858

[B18] Chalazonitis A. D’Autreaux F. Pham T. D. Kessler J. A. Gershon M. D. (2011). Bone morphogenetic proteins regulate enteric gliogenesis by modulating ErbB3 signaling. *Dev. Biol.* 350 64–79 10.1016/j.ydbio.2010.11.017 21094638 PMC3034360

[B19] Chang C. Holtzman D. A. Chau S. Chickering T. Woolf E. A. Holmgren L. M. (2001). Twisted gastrulation can function as a BMP antagonist. *Nature* 410 483–487 10.1038/35068583 11260717

[B20] Chen D. Zhao M. Mundy G. R. (2004). Bone morphogenetic proteins. *Growth Factors* 22 233–241 10.1080/08977190412331279890 15621726

[B21] Chen H. L. Panchision D. M. (2007). Concise review: bone morphogenetic protein pleiotropism in neural stem cells and their derivatives – alternative pathways, convergent signals. *Stem Cells* 25 63–68 10.1634/stemcells.2006-0339 16973830

[B22] Cheng X. Wang Y. He Q. Qiu M. Whittemore S. R. Cao Q. (2007). Bone morphogenetic protein signaling and olig1/2 interact to regulate the differentiation and maturation of adult oligodendrocyte precursor cells. *Stem Cells* 25 3204–3214 10.1634/stemcells.2007-0284 17872503 PMC2742907

[B23] Chmielnicki E. Benraiss A. Economides A. N. Goldman S. A. (2004). Adenovirally expressed noggin and brain-derived neurotrophic factor cooperate to induce new medium spiny neurons from resident progenitor cells in the adult striatal ventricular zone. *J. Neurosci.* 24 2133–2142 10.1523/JNEUROSCI.1554-03.2004 14999064 PMC6730416

[B24] Choe Y. Siegenthaler J. A. Pleasure S. J. (2012). A cascade of morphogenic signaling initiated by the meninges controls corpus callosum formation. *Neuron* 73 698–712 10.1016/j.neuron.2011.11.036 22365545 PMC3292762

[B25] Colak D. Mori T. Brill M. S. Pfeifer A. Falk S. Deng C. (2008). Adult neurogenesis requires Smad4-mediated bone morphogenic protein signaling in stem cells. *J. Neurosci.* 28 434–446 10.1523/JNEUROSCI.4374-07.2008 18184786 PMC6670509

[B26] Collins C. A. DiAntonio A. (2007). Synaptic development: insights from *Drosophila*. *Curr. Opin. Neurobiol.* 17 35–42 10.1016/j.conb.2007.01.001 17229568

[B27] Cui Y. Jean F. Thomas G. Christian J. L. (1998). BMP-4 is proteolytically activated by furin and/or PC6 during vertebrate embryonic development. *EMBO J.* 17 4735–4743 10.1093/emboj/17.16.4735 9707432 PMC1170802

[B28] Derynck R. Zhang Y. E. (2003). Smad-dependent and Smad-independent pathways in TGF-beta family signalling. *Nature* 425 577–584 10.1038/nature02006 14534577

[B29] Doan L. T. Javier A. L. Furr N. M. Nguyen K. L. Cho K. W. Monuki E. S. (2012). A Bmp reporter with ultrasensitive characteristics reveals that high Bmp signaling is not required for cortical hem fate. *PLoS ONE* 7: e44009 10.1371/journal.pone.0044009 PMC3439469 22984456

[B30] Eaton B. A. Davis G. W. (2005). LIM Kinase1 controls synaptic stability downstream of the type II BMP receptor. *Neuron* 47 695–708 10.1016/j.neuron.2005.08.010 16129399

[B31] Ebendal T. Bengtsson H. Soderstrom S. (1998). Bone morphogenetic proteins and their receptors: potential functions in the brain. *J. Neurosci. Res.* 51 139–146 10.1002/(SICI)1097-4547(19980115)51:2 9469567

[B32] Ehrlich M. Gutman O. Knaus P. Henis Y. I. (2012). Oligomeric interactions of TGF-beta and BMP receptors. *FEBS Lett.* 586 1885–1896 10.1016/j.febslet.2012.01.040 22293501

[B33] Eldar A. Dorfman R. Weiss D. Ashe H. Shilo B. Z. Barkai N. (2002). Robustness of the BMP morphogen gradient in *Drosophila* embryonic patterning. *Nature* 419 304–308 10.1038/nature01061 12239569

[B34] Feng X. H. Derynck R. (1997). A kinase subdomain of transforming growth factor-beta (TGF-beta) type I receptor determines the TGF-beta intracellular signaling specificity. *EMBO J.* 16 3912–3923 10.1093/emboj/16.13.3912 9233801 PMC1170015

[B35] Feng X. H. Derynck R. (2005). Specificity and versatility in TGF-β signaling through Smads. *Annu. Rev. Cell Dev. Biol.* 21 659–693 10.1146/annurev.cellbio.21.022404.142018 16212511

[B36] Fernandes M. Antoine M. Hebert J. M. (2012). SMAD4 is essential for generating subtypes of neurons during cerebellar development. *Dev. Biol.* 365 82–90 10.1016/j.ydbio.2012.02.017 22370000 PMC3322275

[B37] Fernandes M. Gutin G. Alcorn H. McConnell S. K. Hebert J. M. (2007). Mutations in the BMP pathway in mice support the existence of two molecular classes of holoprosencephaly. *Development* 134 3789–3794 10.1242/dev.004325 17913790

[B38] Foletta V. C. Lim M. A. Soosairajah J. Kelly A. P. Stanley E. G. Shannon M. (2003). Direct signaling by the BMP type II receptor via the cytoskeletal regulator LIMK1. *J. Cell Biol.* 162 1089–1098 10.1083/jcb.200212060 12963706 PMC2172847

[B39] Fuentealba L. C. Eivers E. Ikeda A. Hurtado C. Kuroda H. Pera E. M. (2007). Integrating patterning signals: Wnt/GSK3 regulates the duration of the BMP/Smad1 signal. *Cell* 131 980–993 10.1016/j.cell.2007.09.027 18045539 PMC2200633

[B40] Fuentes-Medel Y. Ashley J. Barria R. Maloney R. Freeman M. Budnik V. (2012). Integration of a retrograde signal during synapse formation by glia-secreted TGF-beta ligand. *Curr. Biol.* 22 1831–1838 10.1016/j.cub.2012.07.063 22959350 PMC3605899

[B41] Fukuda S. Abematsu M. Mori H. Yanagisawa M. Kagawa T. Nakashima K. (2007). Potentiation of astrogliogenesis by STAT3-mediated activation of bone morphogenetic protein-Smad signaling in neural stem cells. *Mol. Cell. Biol.* 27 4931–4937 10.1128/MCB.02435-06 17452461 PMC1951480

[B42] Furuta Y. Piston D. W. Hogan B. L. (1997). Bone morphogenetic proteins (BMPs) as regulators of dorsal forebrain development. *Development* 124 2203–2212 9187146 10.1242/dev.124.11.2203

[B43] Gamell C. Osses N. Bartrons R. Ruckle T. Camps M. Rosa J. L. (2008). BMP2 induction of actin cytoskeleton reorganization and cell migration requires PI3-kinase and Cdc42 activity. *J. Cell Sci.* 121 3960–3970 10.1242/jcs.031286 19001503

[B44] Gamell C. Susperregui A. G. Bernard O. Rosa J. L. Ventura F. (2011). The p38/MK2/Hsp25 pathway is required for BMP-2-induced cell migration. *PLoS ONE* 6: e16477 10.1371/journal.pone.0016477 PMC3030584 21297993

[B45] Groppe J. Greenwald J. Wiater E. Rodriguez-Leon J. Economides A. N. Kwiatkowski W. (2002). Structural basis of BMP signalling inhibition by the cystine knot protein Noggin. *Nature* 420 636–642 10.1038/nature01245 12478285

[B46] Gross R. E. Mehler M. F. Mabie P. C. Zang Z. Santschi L. Kessler J. A. (1996). Bone morphogenetic proteins promote astroglial lineage commitment by mammalian subventricular zone progenitor cells. *Neuron* 17 595–606 10.1016/S0896-6273(00)80193-2 8893018

[B47] Hall A. K. Miller R. H. (2004). Emerging roles for bone morphogenetic proteins in central nervous system glial biology. *J. Neurosci. Res.* 76 1–8 10.1002/jnr.20019 15048925

[B48] Henriquez J. P. Krull C. E. Osses N. (2011). The Wnt and BMP families of signaling morphogens at the vertebrate neuromuscular junction. *Int. J. Mol. Sci.* 12 8924–8946 10.3390/ijms12128924 22272112 PMC3257109

[B49] Hill C. S. (2009). Nucleocytoplasmic shuttling of Smad proteins. *Cell Res.* 19 36–46 10.1038/cr.2008.325 19114992

[B50] Hinck A. P. (2012). Structural studies of the TGF-betas and their receptors – insights into evolution of the TGF-beta superfamily. *FEBS Lett.* 586 1860–1870 10.1016/j.febslet.2012.05.028 22651914

[B51] Hsieh J. (2012). Orchestrating transcriptional control of adult neurogenesis. *Genes Dev.* 26 1010–1021 10.1101/gad.187336.112 22588716 PMC3360557

[B52] Imayoshi I. Shimogori T. Ohtsuka T. Kageyama R. (2008). Hes genes and neurogenin regulate non-neural versus neural fate specification in the dorsal telencephalic midline. *Development* 135 2531–2541 10.1242/dev.021535 18579678

[B53] Ji S. J. Jaffrey S. R. (2012). Intra-axonal translation of SMAD1/5/8 mediates retrograde regulation of trigeminal ganglia subtype specification. *Neuron* 74 95–107 10.1016/j.neuron.2012.02.022 22500633 PMC3328135

[B54] Kishigami S. Mishina Y. (2005). BMP signaling and early embryonic patterning. *Cytokine Growth Factor Rev.* 16 265–278 10.1016/j.cytogfr.2005.04.002 15871922

[B55] Kohyama J. Sanosaka T. Tokunaga A. Takatsuka E. Tsujimura K. Okano H. (2010). BMP-induced REST regulates the establishment and maintenance of astrocytic identity. *J. Cell Biol.* 189 159–170 10.1083/jcb.200908048 20351065 PMC2854381

[B56] Lee J. Son M. J. Woolard K. Donin N. M. Li A. Cheng C. H. (2008). Epigenetic-mediated dysfunction of the bone morphogenetic protein pathway inhibits differentiation of glioblastoma-initiating cells. *Cancer Cell* 13 69–80 10.1016/j.ccr.2007.12.005 18167341 PMC2835498

[B57] Lee-Hoeflich S. T. Causing C. G. Podkowa M. Zhao X. Wrana J. L. Attisano L. (2004). Activation of LIMK1 by binding to the BMP receptor, BMPRII, regulates BMP-dependent dendritogenesis. *EMBO J.* 23 4792–4801 10.1038/sj.emboj.7600418 15538389 PMC535083

[B58] Le Roux P. Behar S. Higgins D. Charette M. (1999). OP-1 enhances dendritic growth from cerebral cortical neurons in vitro. *Exp. Neurol.* 160 151–163 10.1006/exnr.1999.7194 10630200

[B59] Li H. Richardson W. D. (2008). The evolution of Olig genes and their roles in myelination. *Neuron Glia Biol.* 4 129–135 10.1017/S1740925X09990251 19737433 PMC6326352

[B60] Ligon K. L. Kesari S. Kitada M. Sun T. Arnett H. A. Alberta J. A. (2006). Development of NG2 neural progenitor cells requires Olig gene function. *Proc. Natl. Acad. Sci. U.S.A.* 103 7853–7858 10.1073/pnas.0511001103 16682644 PMC1472534

[B61] Lim D. A. Tramontin A. D. Trevejo J. M. Herrera D. G. Garcia-Verdugo J. M. Alvarez-Buylla A. (2000). Noggin antagonizes BMP signaling to create a niche for adult neurogenesis. *Neuron* 28 713–726 10.1016/S0896-6273(00)00148-3 11163261

[B62] Little S. C. Mullins M. C. (2009). Bone morphogenetic protein heterodimers assemble heteromeric type I receptor complexes to pattern the dorsoventral axis. *Nat. Cell Biol.* 11 637–643 10.1038/ncb1870 19377468 PMC2757091

[B63] Liu A. Niswander L. A. (2005). Bone morphogenetic protein signalling and vertebrate nervous system development. *Nat. Rev. Neurosci.* 6 945–954 16340955 10.1038/nrn1805

[B64] Liu F. Ventura F. Doody J. Massague J. (1995). Human type II receptor for bone morphogenic proteins (BMPs): extension of the two-kinase receptor model to the BMPs. *Mol. Cell. Biol.* 15 3479–3486 10.1038/nrn1805 7791754 PMC230584

[B65] Lu Q. R. Sun T. Zhu Z. Ma N. Garcia M. Stiles C. D. (2002). Common developmental requirement for Olig function indicates a motor neuron/oligodendrocyte connection. *Cell* 109 75–86 10.1016/S0092-8674(02)00678-5 11955448

[B66] Ma C. H. Brenner G. J. Omura T. Samad O. A. Costigan M. Inquimbert P. (2011). The BMP coreceptor RGMb promotes while the endogenous BMP antagonist noggin reduces neurite outgrowth and peripheral nerve regeneration by modulating BMP signaling. *J. Neurosci.* 31 18391–18400 10.1523/JNEUROSCI.4550-11.2011 22171041 PMC3243947

[B67] Mabie P. C. Mehler M. F. Kessler J. A. (1999). Multiple roles of bone morphogenetic protein signaling in the regulation of cortical cell number and phenotype. *J. Neurosci.* 19 7077–7088 10436062 10.1523/JNEUROSCI.19-16-07077.1999 PMC6782885

[B68] Marques G. Bao H. Haerry T. E. Shimell M. J. Duchek P. Zhang B. (2002). The *Drosophila* BMP type II receptor wishful thinking regulates neuromuscular synapse morphology and function. *Neuron* 33 529–543 10.1016/S0896-6273(02)00595-0 11856528

[B69] Marques G. Haerry T. E. Crotty M. L. Xue M. Zhang B O’Connor M. B. (2003). Retrograde Gbb signaling through the Bmp type 2 receptor wishful thinking regulates systemic FMRFa expression in *Drosophila*. *Development* 130 5457–5470 10.1242/dev.00772 14507784

[B70] Matsuura I. Taniguchi J. Hata K. Saeki N. Yamashita T. (2008). BMP inhibition enhances axonal growth and functional recovery after spinal cord injury. *J. Neurochem.* 105 1471–1479 10.1111/j.1471-4159.2008.05251.x 18221366

[B71] McCabe B. D. Marques G. Haghighi A. P. Fetter R. D. Crotty M. L. Haerry T. E. (2003). The BMP homolog Gbb provides a retrograde signal that regulates synaptic growth at the *Drosophila* neuromuscular junction. *Neuron* 39 241–254 10.1016/S0896-6273(03)00426-4 12873382

[B72] Mehler M. F. (2002). Mechanisms regulating lineage diversity during mammalian cerebral cortical neurogenesis and gliogenesis. *Results Probl. Cell Differ.* 39 27–52 10.1007/978-3-540-46006-0_2 12357985

[B73] Mehler M. F. Mabie P. C. Zhang D. Kessler J. A. (1997). Bone morphogenetic proteins in the nervous system. *Trends Neurosci.* 20 309–317 10.1016/S0166-2236(96)01046-6 9223224

[B74] Mekki-Dauriac S. Agius E. Kan P. Cochard P. (2002). Bone morphogenetic proteins negatively control oligodendrocyte precursor specification in the chick spinal cord. *Development* 129 5117–5130 12399304 10.1242/dev.129.22.5117

[B75] Meynard D. Kautz L. Darnaud V. Canonne-Hergaux F. Coppin H. Roth M. P. (2009). Lack of the bone morphogenetic protein BMP6 induces massive iron overload. *Nat. Genet.* 41 478–481 10.1038/ng.320 19252488

[B76] Mira H. Andreu Z. Suh H. Lie D. C. Jessberger S. Consiglio A. (2010). Signaling through BMPR-IA regulates quiescence and long-term activity of neural stem cells in the adult hippocampus. *Cell Stem Cell* 7 78–89 10.1016/j.stem.2010.04.016 20621052

[B77] Miyagi M. Mikawa S. Hasegawa T. Kobayashi S. Matsuyama Y. Sato K. (2011). Bone morphogenetic protein receptor expressions in the adult rat brain. *Neuroscience* 176 93–109 10.1016/j.neuroscience.2010.12.027 21185359

[B78] Miyazono K. Kamiya Y. Morikawa M. (2010). Bone morphogenetic protein receptors and signal transduction. *J. Biochem.* 147 35–51 10.1093/jb/mvp148 19762341

[B79] Mueller T. D. Nickel J. (2012). Promiscuity and specificity in BMP receptor activation. *FEBS Lett.* 586 1846–1859 10.1016/j.febslet.2012.02.043 22710174

[B80] Nakashima K. Yanagisawa M. Arakawa H. Kimura N. Hisatsune T. Kawabata M. (1999). Synergistic signaling in fetal brain by STAT3-Smad1 complex bridged by p300. *Science* 284 479–482 10.1126/science.284.5413.479 10205054

[B81] Norton J. D. (2000). ID helix-loop-helix proteins in cell growth, differentiation and tumorigenesis. *J. Cell Sci.* 113(Pt 22) 3897–3905 11058077 10.1242/jcs.113.22.3897

[B82] Onichtchouk D. Chen Y. G. Dosch R. Gawantka V. Delius H. Massague J. (1999). Silencing of TGF-beta signalling by the pseudoreceptor BAMBI. *Nature* 401 480–485 10.1038/46794 10519551

[B83] Osses N. Gutierrez J. Lopez-Rovira T. Ventura F. Brandan E. (2006). Sulfation is required for bone morphogenetic protein 2-dependent Id1 induction. *Biochem. Biophys. Res. Commun.* 344 1207–1215 10.1016/j.bbrc.2006.04.029 16647687

[B84] Peluso C. E. Umulis D. Kim Y. J. O’Connor M. B. Serpe M. (2011). Shaping BMP morphogen gradients through enzyme-substrate interactions. *Dev. Cell* 21 375–383 10.1016/j.devcel.2011.06.025 21839924 PMC3175245

[B85] Perron J. C. Dodd J. (2011). Inductive specification and axonal orientation of spinal neurons mediated by divergent bone morphogenetic protein signaling pathways. *Neural Dev.* 6 36 10.1186/1749-8104-6-36 PMC3227570 22085733

[B86] Perron J. C. Dodd J. (2012). Structural distinctions in BMPs underlie divergent signaling in spinal neurons. *Neural Dev.* 7 16 10.1186/1749-8104-7-16 PMC3403000 22559862

[B87] Piccirillo S. G. Reynolds B. A. Zanetti N. Lamorte G. Binda E. Broggi G. (2006). Bone morphogenetic proteins inhibit the tumorigenic potential of human brain tumour-initiating cells. *Nature* 444 761–765 10.1038/nature05349 17151667

[B88] Podkowa M. Zhao X. Chow C. W. Coffey E. T. Davis R. J. Attisano L. (2010). Microtubule stabilization by bone morphogenetic protein receptor-mediated scaffolding of c-Jun N-terminal kinase promotes dendrite formation. *Mol. Cell. Biol.* 30 2241–2250 10.1128/MCB.01166-09 20176805 PMC2863594

[B89] Rajan P. Panchision D. M. Newell L. F. McKay R. D. (2003). BMPs signal alternately through a SMAD or FRAP-STAT pathway to regulate fate choice in CNS stem cells. *J. Cell Biol.* 161 911–921 10.1083/jcb.200211021 12796477 PMC2172962

[B90] Ramel M. C. Hill C. S. (2012). Spatial regulation of BMP activity. *FEBS Lett.* 586 1929–1941 10.1016/j.febslet.2012.02.035 22710177

[B91] Rawson J. M. Lee M. Kennedy E. L. Selleck S. B. (2003). *Drosophila* neuromuscular synapse assembly and function require the TGF-beta type I receptor saxophone and the transcription factor Mad. *J. Neurobiol.* 55 134–150 10.1002/neu.10189 12672013

[B92] Rider C. C. Mulloy B. (2010). Bone morphogenetic protein and growth differentiation factor cytokine families and their protein antagonists. *Biochem. J.* 429 1–12 10.1042/BJ20100305 20545624

[B93] Ross J. J. Shimmi O. Vilmos P. Petryk A. Kim H. Gaudenz K. (2001). Twisted gastrulation is a conserved extracellular BMP antagonist. *Nature* 410 479–483 10.1038/35068578 11260716

[B94] Ross S. E. Greenberg M. E. Stiles C. D. (2003). Basic helix-loop-helix factors in cortical development. *Neuron* 39 13–25 10.1016/S0896-6273(03)00365-9 12848929

[B95] Ruppert R. Hoffmann E. Sebald W. (1996). Human bone morphogenetic protein 2 contains a heparin-binding site which modifies its biological activity. *Eur. J. Biochem.* 237 295–302 10.1111/j.1432-1033.1996.0295n.x 8620887

[B96] Ruzinova M. B. Benezra R. (2003). Id proteins in development, cell cycle and cancer. *Trends Cell Biol.* 13 410–418 10.1016/S0962-8924(03)00147-8 12888293

[B97] Sabo J. K. Aumann T. D. Merlo D. Kilpatrick T. J. Cate H. S. (2011). Remyelination is altered by bone morphogenic protein signaling in demyelinated lesions. *J. Neurosci.* 31 4504–4510 10.1523/JNEUROSCI.5859-10.2011 21430151 PMC6622914

[B98] Sabo J. K. Cate H. S. (2013). Signalling pathways that inhibit the capacity of precursor cells for myelin repair. *Int. J. Mol. Sci.* 14 1031–1049 10.3390/ijms14011031 23296277 PMC3565305

[B99] Sabo J. K. Kilpatrick T. J. Cate H. S. (2009). Effects of bone morphogenic proteins on neural precursor cells and regulation during central nervous system injury. *Neurosignals* 17 255–264 10.1159/000231892 19816062

[B100] Samanta J. Kessler J. A. (2004). Interactions between ID and OLIG proteins mediate the inhibitory effects of BMP4 on oligodendroglial differentiation. *Development* 131 4131–4142 10.1242/dev.01273 15280210

[B101] Sanchez-Camacho C. Bovolenta P. (2009). Emerging mechanisms in morphogen-mediated axon guidance. *Bioessays* 31 1013–1025 10.1002/bies.200900063 19705365

[B102] Sanchez-Camacho C. Ortega J. A. Ocana I. Alcantara S. Bovolenta P. (2011). Appropriate Bmp7 levels are required for the differentiation of midline guidepost cells involved in corpus callosum formation. *Dev. Neurobiol.* 71 337–350 10.1002/dneu.20865 21485009

[B103] Saremba S. Nickel J. Seher A. Kotzsch A. Sebald W. Mueller T. D. (2008). Type I receptor binding of bone morphogenetic protein 6 is dependent on N-glycosylation of the ligand. *FEBS J.* 275 172–183 10.1111/j.1742-4658.2007.06187.x 18070108

[B104] Sawala A. Sutcliffe C. Ashe H. L. (2012). Multistep molecular mechanism for bone morphogenetic protein extracellular transport in the *Drosophila* embryo. *Proc. Natl. Acad. Sci. U.S.A.* 109 11222–11227 10.1073/pnas.1202781109 22733779 PMC3396515

[B105] Scheufler C. Sebald W. Hulsmeyer M. (1999). Crystal structure of human bone morphogenetic protein-2 at 2.7 A resolution. *J. Mol. Biol.* 287 103–115 10.1006/jmbi.1999.2590 10074410

[B106] Scholl C. Weibetamuller K. Holenya P. Shaked-Rabi M. Tucker K. L. Wolfl S. (2012). Distinct and overlapping gene regulatory networks in BMP- and HDAC-controlled cell fate determination in the embryonic forebrain. *BMC Genomics* 13: 298 10.1186/1471-2164-13-298 PMC3460768 22748179

[B107] See J. Mamontov P. Ahn K. Wine-Lee L. Crenshaw E. B. III Grinspan J. B. (2007). BMP signaling mutant mice exhibit glial cell maturation defects. *Mol. Cell. Neurosci.* 35 171–182 10.1016/j.mcn.2007.02.012 17391983 PMC1950488

[B108] See J. M. Grinspan J. B. (2009). Sending mixed signals: bone morphogenetic protein in myelination and demyelination. *J. Neuropathol. Exp. Neurol.* 68 595–604 10.1097/NEN.0b013e3181a66ad9 19458544

[B109] Shi Y. Hata A. Lo R. S. Massague J. Pavletich N. P. (1997). A structural basis for mutational inactivation of the tumour suppressor Smad4. *Nature* 388 87–93 10.1038/40431 9214508

[B110] Shi Y. Massague J. (2003). Mechanisms of TGF-beta signaling from cell membrane to the nucleus. *Cell* 113 685–700 10.1016/S0092-8674(03)00432-X 12809600

[B111] Sieber C. Kopf J. Hiepen C. Knaus P. (2009). Recent advances in BMP receptor signaling. *Cytokine Growth Factor Rev.* 20 343–355 10.1016/j.cytogfr.2009.10.007 19897402

[B112] Sim F. J. Lang J. K. Waldau B. Roy N. S. Schwartz T. E. Pilcher W. H. (2006). Complementary patterns of gene expression by human oligodendrocyte progenitors and their environment predict determinants of progenitor maintenance and differentiation. *Ann. Neurol.* 59 763–779 10.1002/ana.20812 16634042

[B113] Smith R. B. Machamer J. B. Kim N. C. Hays T. S. Marques G. (2012). Relay of retrograde synaptogenic signals through axonal transport of BMP receptors. *J. Cell Sci.* 125 3752–3764 10.1242/jcs.094292 22573823 PMC3462079

[B114] Sorrentino A. Thakur N. Grimsby S. Marcusson A. von Bulow V. Schuster N. (2008). The type I TGF-beta receptor engages TRAF6 to activate TAK1 in a receptor kinase-independent manner. *Nat. Cell Biol.* 10 1199–1207 10.1038/ncb1780 18758450

[B115] Sun Y. Hu J. Zhou L. Pollard S. M. Smith A. (2011). Interplay between FGF2 and BMP controls the self-renewal, dormancy and differentiation of rat neural stem cells. *J. Cell Sci.* 124 1867–1877 10.1242/jcs.085506 21558414 PMC3096055

[B116] Susperregui A. R. Gamell C. Rodriguez-Carballo E. Ortuno M. J. Bartrons R. Rosa J. L. (2011). Noncanonical BMP signaling regulates cyclooxygenase-2 transcription. *Mol. Endocrinol.* 25 1006–1017 10.1210/me.2010-0515 21436263 PMC5417253

[B117] Takizawa T. Ochiai W. Nakashima K. Taga T. (2003). Enhanced gene activation by Notch and BMP signaling cross-talk. *Nucleic Acids Res.* 31 5723–5731 10.1093/nar/gkg778 14500836 PMC206475

[B118] Ulsamer A. Ortuno M. J. Ruiz S. Susperregui A. R. Osses N. Rosa J. L. (2008). BMP-2 induces Osterix expression through up-regulation of Dlx5 and its phosphorylation by p38. *J. Biol. Chem.* 283 3816–3826 10.1074/jbc.M704724200 18056716

[B119] Urist M. R. (1965). Bone: formation by autoinduction. *Science* 150 893–899 10.1126/science.150.3698.893 5319761

[B120] Valera E. Isaacs M. J. Kawakami Y. Izpisua Belmonte J. C. Choe S. (2010). BMP-2/6 heterodimer is more effective than BMP-2 or BMP-6 homodimers as inductor of differentiation of human embryonic stem cells. *PLoS ONE* 5: e11167 10.1371/journal.pone.0011167 PMC2887366 20567515

[B121] Vinals F. Reiriz J. Ambrosio S. Bartrons R. Rosa J. L. Ventura F. (2004). BMP-2 decreases Mash1 stability by increasing Id1 expression. *EMBO J.* 23 3527–3537 10.1038/sj.emboj.7600360 15318167 PMC516632

[B122] Wagner D. O. Sieber C. Bhushan R. Borgermann J. H. Graf D. Knaus P. (2010). BMPs: from bone to body morphogenetic proteins. *Sci. Signal.* 3 mr1 10.1126/scisignal.3107mr1 20124549

[B123] Walsh D. W. Godson C. Brazil D. P. Martin F. (2010). Extracellular BMP-antagonist regulation in development and disease: tied up in knots. *Trends Cell Biol.* 20 244–256 10.1016/j.tcb.2010.01.008 20188563

[B124] Wen Z. Han L. Bamburg J. R. Shim S. Ming G. L. Zheng J. Q. (2007). BMP gradients steer nerve growth cones by a balancing act of LIM kinase and Slingshot phosphatase on ADF/cofilin. *J. Cell Biol.* 178 107–119 10.1083/jcb.200703055 17606869 PMC2064427

[B125] Weng Q. Chen Y. Wang H. Xu X. Yang B. He Q. (2012). Dual-mode modulation of Smad signaling by Smad-interacting protein Sip1 is required for myelination in the central nervous system. *Neuron* 73 713–728 10.1016/j.neuron.2011.12.021 22365546 PMC3293152

[B126] Wicks S. J. Grocott T. Haros K. Maillard M. ten Dijke P. Chantry A. (2006). Reversible ubiquitination regulates the Smad/TGF-beta signalling pathway. *Biochem. Soc. Trans.* 34 761–763 10.1042/BST0340761 17052192

[B127] Wieser R. Wrana J. L. Massague J. (1995). GS domain mutations that constitutively activate T beta R-I, the downstream signaling component in the TGF-beta receptor complex. *EMBO J.* 14 2199–2208 7774578 10.1002/j.1460-2075.1995.tb07214.x PMC398326

[B128] Withers G. S. Higgins D. Charette M. Banker G. (2000). Bone morphogenetic protein-7 enhances dendritic growth and receptivity to innervation in cultured hippocampal neurons. *Eur. J. Neurosci.* 12 106–116 10.1046/j.1460-9568.2000.00889.x 10651865

[B129] Wrana J. L. Attisano L. Wieser R. Ventura F. Massague J. (1994). Mechanism of activation of the TGF-beta receptor. *Nature* 370 341–347 10.1038/370341a0 8047140

[B130] Wu M. Hernandez M. Shen S. Sabo J. K. Kelkar D. Wang J. (2012). Differential modulation of the oligodendrocyte transcriptome by sonic hedgehog and bone morphogenetic protein 4 via opposing effects on histone acetylation. *J. Neurosci.* 32 6651–6664 10.1523/JNEUROSCI.4876-11.2012 22573687 PMC3412138

[B131] Xia Y. Yu P. B. Sidis Y. Beppu H. Bloch K. D. Schneyer A. L. (2007). Repulsive guidance molecule RGMa alters utilization of bone morphogenetic protein (BMP) type II receptors by BMP2 and BMP4. *J. Biol. Chem.* 282 18129–18140 10.1074/jbc.M701679200 17472960

[B132] Xu X. Han J. Ito Y. Bringas P. Jr. Deng C. Chai Y. (2008). Ectodermal Smad4 and p38 MAPK are functionally redundant in mediating TGF-beta/BMP signaling during tooth and palate development. *Dev. Cell* 15 322–329 10.1016/j.devcel.2008.06.004 18694570 PMC2610417

[B133] Yamashita M. Fatyol K. Jin C. Wang X. Liu Z. Zhang Y. E. (2008). TRAF6 mediates Smad-independent activation of JNK and p38 by TGF-beta. *Mol. Cell* 31 918–924 10.1016/j.molcel.2008.09.002 18922473 PMC2621323

[B134] Yamauchi K. Phan K. D. Butler S. J. (2008). BMP type I receptor complexes have distinct activities mediating cell fate and axon guidance decisions. *Development* 135 1119–1128 10.1242/dev.012989 18272594

[B135] Yamauchi K. Varadarajan S. G. Li J. E. Butler S. J. (2013). Type Ib BMP receptors mediate the rate of commissural axon extension through inhibition of cofilin activity. *Development* 140 333–342 10.1242/dev.089524 23250207 PMC3597210

[B136] Yi S. E. Daluiski A. Pederson R. Rosen V. Lyons K. M. (2000). The type I BMP receptor BMPRIB is required for chondrogenesis in the mouse limb. *Development* 127 621–630 10631182 10.1242/dev.127.3.621

[B137] Zakin L De Robertis E. M. (2010). Extracellular regulation of BMP signaling. *Curr. Biol.* 20 R89–R92 10.1016/j.cub.2009.11.021 20144774 PMC3034644

[B138] Zhang Y. E. (2009). Non-Smad pathways in TGF-beta signaling. *Cell Res.* 19 128–139 10.1038/cr.2008.328 19114990 PMC2635127

